# Medical History from the Earliest Times

**Published:** 1892-12-03

**Authors:** E. A. Withington


### Medical History from the Earliest Times.

By E. T. Withington, M.A., M.B. Oxon.

### XXXIV.—The Darkest Age.

Many people object to the epithet "dark" as applied to the Middle Ages, and some have even wished themselves back in that romantic epoch of chivalry and troubadours, crusades, and cathedral building; but there can be little doubt of the propriety of the term when used to indicate the state of Western Europe from the fifth to the eleventh century. Science and literature, if we may use those words at all, were confined to the clergy, and how little they possessed the following facts will show. A Spanish synod of the seventh century decreed that no one should receive priest's orders in future unless he could at least read the Psalms and the baptismal service. In Italy, about the same time, a patriotic historian can find only three schools, and he doubts whether much more than bad reading was taught at any of them. France was still more unfortunate, for we are told that under Charles Martel what schools remained were presided over by discharged soldiers, who could neither read nor write. England was a brilliant exception. Archbishop Theodore had brought with him from Tarsus a Greek library, and the school of Canterbury produced men who could read Homer, and, what is more to our purpose, could translate Dioscorides. But with this exception Greek was unknown in Western Europe, and Greek was still the key to all higher knowledge, especially in medicine. Latin versions of some Galenic treatises, indeed, existed, but they were so little known that Constantine the African could boast in the eleventh century that he was the first to translate that author. Celsus, whose work might have formed an excellent text-book, was almost entirely forgotten, and though Cassiodorus urged his monks to read the Methodic compendium of Caelius Aurelianus, the extreme rarity of the manuscripts indicates that his advice was seldom followed.

The Empiric school reigned without a rival, but that great medical sect had sadly degenerated since the days of Heraclides. Two legs of its famous "tripod" had given way altogether, for the later Empirics were incapable of scientific "observation," and would not have understood the meaning of "analogy." "History" still remained, but was chiefly comprised in the "Natural History" of Pliny the Elder, from which such medical writers as Theodore Priscian, Pliny Valerianus, Quintus Serenus, and Sextus Placitus largely borrowed, preferring always the more marvellous statements of that credulous compiler, and adding a few absurd or disgusting prescriptions of their own. The following examples may suffice: "Asses dung dried and used as a dentifrice," says Priscian, "will immediately cure toothache, and it is equally effectual when mixed with vinegar and held for some time in the mouth." "If a child kisses a horse's nose," declares Sextus Placitus, "he will never have toothache, but take great care the horse does not bite the child." Two members of the school, Apuleius and Marcellus Empiricus, deserve more special consideration, and their works represent the two types on which all the medical books of this period are written. Apuleius gives the names of various herbs, followed by lists of the diseases they will cure, while Marcellus enumerates diseases in order from head to foot, adding their various remedies, magical or rational, but neither pays the least attention to diagnosis, prognosis, or any other division of medical science. The former writer is also interesting as showing the transition from heathen to Christian medicine. Apuleius was a pagan, and in the oldest manuscripts the names of various herbs are followed by prayers and incantations to be recited on gathering them, handed down, perhaps, from the old Greek or Tuscan herbalists; but the monkish transcribers have converted him into a Christian by the simple process of slightly altering these prayers; while, in later editions, they are either omitted altogether—as in the Anglo-Saxon version—or replaced by the Creed and Pater Noster, which the canons of the Church declared might alone be repeated on such occasions.

But the incantations of Apuleius are sense and soberness compared with those of his Christian successor, Marcellus (a.d. 480). "If a man's nose bleeds whisper in his ear on the same side, 'socsocam sykyma' thrice nine times—and you may still go on saying it." Toothache, if it occurs on a Tuesday or Thursday, and if the moon is waning, may be cured by repeating seven times "argidum margidum stargidum." Even his prayers compare unfavourably with those of Apuleius, "In ophthalmia look out for the first swallow, then run silently to the nearest spring, wash your eyes, and pray God that you may be free from it for that year, and that all the pain may pass into the swallow." The whole book is full of similar absurdities, which he defends by the invariable empiric argument that patients have got well after practising them.

We must not, however, suppose that this superstition was in any way favoured by Christianity. It was indeed a Christian Bishop who introduced the disastrous doctrine that the signs of the Zodiac preside over the various organs of the human body, but the Church disapproved both of the science and the theology of Priscillian, and he was executed as a magician and heretic (a.d. 385). Besides condemning magic, the Church further attempted to direct the excessive faith in the supernatural into higher channels. Instead of invoking Juno Lucina, and other heathen demons, the young mother was encouraged to repeat the 18th Psalm, and to think of the wonderful deliverance of St. Margaret, when swallowed by Satan in the form of a dragon. Did a man suffer from toothache, or colic, or pains in the back? Let him, instead of decking himself with pagan amulets, consider how much greater pain of those various kinds was endured by St. Apollonia when all her teeth were knocked out; by St. Erasmus when his bowels were torn from his living body; or by St. Lawrence when he was roasting on the gridiron. And in his prayers for health he would naturally introduce the names of those holy martyrs, or of other saints who were similarly connected with other diseases, with a firm faith in their effectual intercession.

New superstitions, however, soon appeared remarkably resembling those they replaced. Just as plagues and epidemics were universally ascribed to the wrath of God, so particular diseases came to be attributed to the anger of particular saints, while, on the other hand even the images of other saints were accredited with magical powers of healing; the very sight of a picture of St. Christopher, for instance, sufficed to protect a person from sudden death for a whole day, as is evidenced by the ancient doggrel—

O Christophore sancte, virtutes sunt tibi tantae,
Quis te mane videt nocturno tempore ridet.

